# Investigation on Buckling Performance of Prefabricated Light Steel Frame Materials under the Action of Random Defects during Construction

**DOI:** 10.3390/ma16165666

**Published:** 2023-08-17

**Authors:** Gang Yao, Yuxiao Chen, Yang Yang, Xinlong Ma, Wulei Men

**Affiliations:** Key Laboratory of New Technology for Construction of Cities in Mountain Area, School of Civil Engineering, Chongqing University, Chongqing 400045, China

**Keywords:** light steel frame materials, construction stages, random defects, two-factor analysis, numerical simulation

## Abstract

This investigation proposes an analytical approach for analyzing the impact of random defects on light steel frame materials. The addition of random defects for the overall and the component units was achieved by integrating Matlab R2022a and Ansys R19.0 finite element software. Nonlinear analysis was conducted to calculate ultimate load factors and nodal ultimate displacements of the materials under various random defects at each stage of construction. A two-factor analysis was employed to investigate the effects of random defects on the calculation results during different construction stages. The investigation reveals that the response of the light steel frame materials to initial defects is more pronounced during the construction stage. Moreover, the construction stage is the main factor that affects the ultimate load factor and nodal ultimate displacement, compared with random defects. The influence of different random defects on structural displacements varies significantly. The displacement development of the light steel frame materials under the influence of component unit defects tends to be more rapid than that of the overall defects. However, their buckling critical loads are essentially similar.

## 1. Introduction

Steel is an extensively used material in prefabricated buildings due to its lightweight, high strength, ease of production and processing, high construction efficiency, and strong assemblability [1]. Furthermore, under the promotion of the Chinese policy, assembled light steel materials have emerged as a preferred option for rural housing renovation and residential construction and have been built in large numbers [2,3]. Currently, common low-rise assembled building structural systems include the composite insulated reinforced welded mesh concrete shear wall system (CL building structural system), the assembled composite wall structural system, the prefabricated reinforced concrete hollow mould shear wall structural system, the assembled wood structural system, the dense-column-supported frame structural system, the modular structural system, and the low-rise assembled light steel framed structural system [4,5,6,7]. Among these, the low-rise assembled light steel frame structure system is the most favored for low-rise building structures due to its advantages in modularization, standardization, environmentalization, economization, factory assembly, and informationization production.

Congenital, processing, fabrication, connection, transportation, and installation defects in steel structures significantly impact their safety and applicability. Especially in rural construction, with many unique characteristics, light steel structures’ production and installation stages are particularly susceptible to a decline in structural performance due to construction irregularities. Random defects can exacerbate this issue, potentially resulting in premature material buckling and posing a significant risk to the building’s safety. Furthermore, improving the flexural properties of materials under the effect of defects not only contributes to the structure’s safety but also helps save costs and improve construction efficiency. Therefore, the impact of structural defects on prefabricated steel materials cannot be overlooked [8].

Currently, research on the stability and bearing capacity of steel frame materials with defects mostly focuses on large and complex structures, such as mesh frames and mesh shell structures. The defects studied are primarily initial geometrical defects and residual stresses in the structure or members [9,10,11,12,13]. Usually, the initial geometric defects mainly refer to the buckling of the bar, increasing the perturbation and deformation of the material, and the defects’ shape can be simulated by sinusoidal waves [14]. Residual stresses mainly refer to the stresses present in hot-rolled or welded steel members from processes such as rolling, welding, and cold forming, which may reduce the material’s fatigue life and increase the risk of fracture [15]. Kani et al. [16] used the tangent stiffness matrix to solve the instability mode of the structure and found that node misalignment has a more significant effect on the structure than defects in the bars. Bielewicz et al. [17] conducted a stochastic analysis on the static response of a nonlinear model of a defective shell structure, combining the Monte Carlo method with finite element program analysis to analyze the nonlinear post-buckling behavior of shells and discuss the method’s accuracy. Lauterbach et al. [18] used a stochastic method to simulate structural imperfections and found that the effect of geometrical imperfections can be neglected in areas where support exists in thin-walled members. Kala et al. [19] investigated the deformation of planar trusses with stochastic imperfections subjected to vertical loading and found that asymmetric defects have a detrimental effect on the load-carrying capacity of the trusses. Roy et al. investigated the flexural behavior of back-to-back built-up cold-formed steel and the axial strength of angle columns through tests and numerical simulations [20,21].

Research on defects of steel frame materials started earlier and developed faster, and the current focus is on constructing a database of material defects and studying the effects of defects on structures by probabilistic methods. Arrayago et al. [22] conducted a statistical analysis of the main parameters affecting the strength of steel based on data from the last decades. They considered factors such as steel type, cross-section geometry, defects, and residual stresses and proposed a compatible probabilistic model to provide a database for research on steel structure defects. Mirzaie [23] measured the geometry of steel tube defects, analyzed the characteristics of the defects caused by the manufacturing process and the errors in the measurement of the defects, and demonstrated the feasibility of using probabilistic methods to generate geometric defects consistent with the measurements. Fina et al. [24] used a probabilistic approach to establish a Gaussian random field for random defects, extending the classical probabilistic approach to the fuzzy-random approach, providing a more reasonable description of the inaccurate random defect sampling method and evaluating the simulation results of the fit.

In advanced structural analysis, there are three primary methods for considering structural defects: the direct analysis method [25], the equivalent nominal load method [26], and the reduced tangent modulus method [27]. The equivalent nominal load and reduced tangential modulus methods are approximate methods proposed when computer technology was not yet mature [28]. As hardware and software have developed, the direct analysis method of the overall structure has been adopted by design codes, and it is the most commonly used structural analysis method now. The direct analysis method involves introducing a definite defect value directly on the member in the structural analysis [29,30,31]. This method recognizes that the initial state of the member is no longer ideal and considers the direct effect of the defect on the light steel materials.

Research on defects mainly focuses on traditional steel structures, with a limited investigation on the working performance of low-rise light steel materials under defective states [32,33,34]. Additionally, relevant codes have not made provisions for this, and the steel structure design code is still adopted as the defect control standard in the design of light steel structures. Therefore, it is crucial to investigate the effect of initial defects on the stability performance of assembled lightweight steel buildings, given their widespread popularity [35,36]. To ensure the safety of light steel framed buildings and the sustainability of this material, this paper proposes an improved Monte Carlo-based method to analyze the buckling performance of low-rise assembled light steel frame materials under the separate effects of random overall geometrical defects and random component geometrical defects during the structure’s service and construction stages.

## 2. Model of Low-Rise Assembled Light Steel Frames

### 2.1. Project Overview

**Figure 1:**
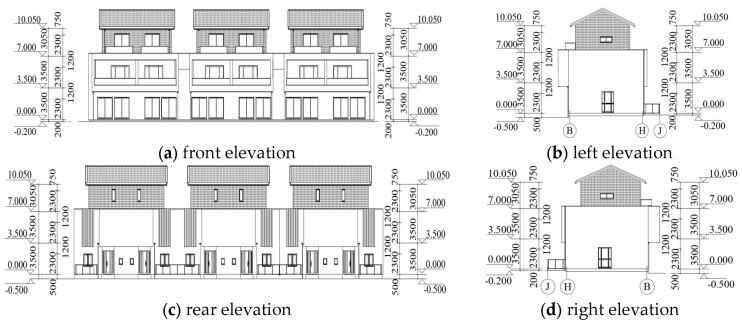
Elevation drawings of the light steel structure.

This study focuses on a residential project in Shangxing Town of Liyang City, as shown in Figure 1. This low-rise steel residence is constructed with light steel frame materials. The project consists of three floors above ground and has a design service life of 50 years. The building is classified as structural safety class II, with a structural importance coefficient of 1.0, a seismic protection category of C, a seismic protection intensity of 7 degrees, and a design basic seismic acceleration of 0.10 g.

According to the Load Code for the Design of Building Structures [37] and the Code for Construction of Steel Structures [38], structural loads are assessed based on the construction and usage stages. For permanent loads, the value remains consistent during the construction and usage stages, whereas variable loads are determined by the actual load conditions of the structure in each stage. Load combinations are established as per the specifications, and due to the significant differences in horizontal stiffness, horizontal loads are combined based on the direction of the structure’s smaller lateral stiffness. Moreover, given the short construction period for light steel structures, seismic action during the construction stage is not considered, and code-specified structural measures are employed to ensure load-carrying capacity. Table 1 displays the structural load conditions, while Table 2 presents the standard values of structural loads [37].

### 2.2. Finite Element Modeling

The project’s beams, columns, purlins, and braces are constructed from Q235-B steel, which satisfies the Von Mises yield criterion. The steel’s modulus of elasticity is 2.06 × 10^5^ MPa, with a Poisson’s ratio of 0.24. The concrete has a modulus of elasticity of 3 × 10^4^ MPa and a Poisson’s ratio of 0.2. Both steel and concrete are isotropic materials.

**Figure 2:**
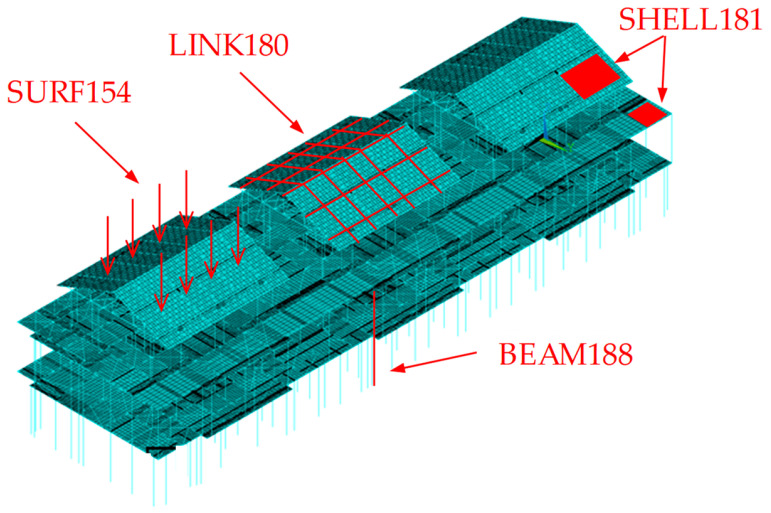
Schematic diagram of the finite element model.

The finite element analysis software Ansys is selected for the structural analysis. BEAM188 cell (3D current finite strain beam cell) was used to simulate the beams, columns, purlins, and bracing, with two integration points set along the length of the beams. The floors and roofs were simulated using the SHELL181 cell (4-node finite strain shell cell), with the cell integration option selecting the full integration of the uncoordinated mode and the bending and membrane stiffness considered. The horizontal support of the structure was simulated using the LINK180 cell (3D finite strain rod cell) [39]. Furthermore, the SURF154 cell (3D structural surface effect cell) overlays on the SHELL cell to apply various loads and surface effects. The components are interconnected through coupling that considers all degrees of freedom, mimicking welded or bolted connections. Moreover, the primary functions of the inner partition wall and exterior walls in the light steel frame synergistic system are space partitioning and enclosure. Consequently, during the modeling process, these elements are considered equivalent to homogeneous loads applied to the beam and column members of the system. The column and beam shapes and dimensions of the structure are shown in Table 3, and the model is shown in Figure 2.

The accuracy of finite element calculations is affected by the mesh size used. Generally, finer mesh sizes yield more accurate results but require longer calculation times, while larger mesh sizes may lead to inaccurate or incorrect results. For eigenvalue buckling analysis, Ansys does not distinguish between local and overall buckling, making it necessary to set the number of mesh divisions in the columns to one to obtain the overall buckling mode of the materials and determine their defective form. To facilitate subsequent analysis, the finite element model based on the ideal materials was meshed, and the maximum displacement and first-order buckling mode were calculated in the model under working condition 1. A suitable finite element model for adding random defects was then selected. The meshing method is shown in Table 4.

**Figure 3:**
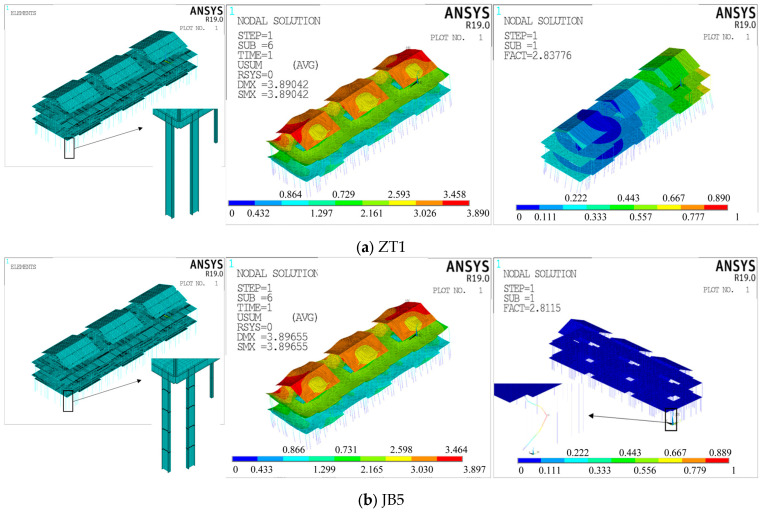
Schematic diagram of the selected finite element model (from left to right: model, static analysis, and eigenvalue buckling analysis).

The meshing method utilized in this study generated 36 sets of data. Trial calculations revealed that the finite element results were more stable when the mesh was divided into smaller cells. Specifically, the first-order buckling factor obtained from the structural eigenvalue buckling analysis was slightly larger when the column mesh was divided into 1, compared to when it was divided into 5 or 10. This was because overall instability occurs when more than one local member is destabilized, and it is difficult to obtain the overall instability pattern of the structure if the column mesh is not divided into 1 [40]. The average deviation of the structural static analysis displacement solution due to changes in the column mesh size was approximately 1 × 10^−5^, while the average deviation of the structural first-order buckling factor was about 1.2 × 10^−3^, which was negligible. Similarly, when the beam mesh division was changed, the average deviation of the structural static analysis displacement solution was about 1.5%, and the average deviation of the structural first-order buckling factor was about 1.2 × 10^−3^. However, when the plate mesh division was finer, the deviation of the two solutions was below 1 × 10^−4^, and its effect was negligible. It is worth noting that the mesh division of the plate is associated with the beam, and the computational accuracy increases with finer divisions. However, the number of cells and the size of the model file also increase significantly. Considering the balance between computational accuracy and model file size, selected model number ZT1 for overall random defect analysis and model number JB5 for component unit random defects analysis. Table 5 and Figure 3 show the selected finite element model and calculation results.

**Figure 4:**
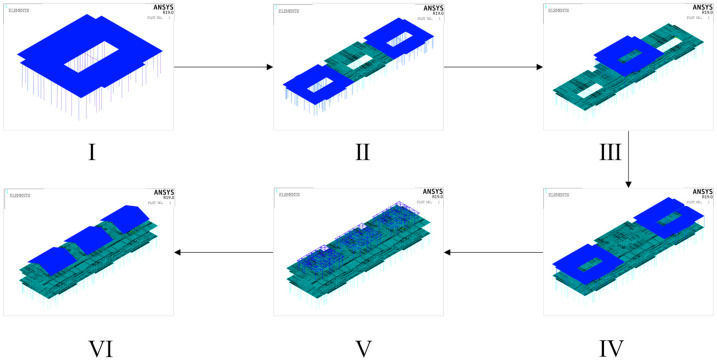
Schematic illustration of construction stages division.

The simulation of the construction stage was conducted using the elastic-plastic stress analysis with the death/birth elements method, which utilizes the EKILL and EALIVE commands to activate or deactivate corresponding units [41]. However, in Ansys, the EKILL command does not delete the unit but reduces its stiffness to a small value. Consequently, the killed unit still participates in the calculation, necessitating further constraints on the nodes of the killed unit to ensure calculation accuracy. In this investigation, the light steel frame structure was divided into six construction stages based on the degree of completion, following the principle of constructing from the bottom to the top and from the middle to both sides. The construction stages were as follows: the bottom intermediate frame, the bottom frame, the two-story intermediate frame, the two-story frame, the roof beams, and the roof panels. The finite element model is shown in Figure 4, with the blue section representing the newly constructed materials in the current construction stage.

### 2.3. Determination of the Most Unfavorable Working Conditions

To determine the most unfavorable working conditions under the four loads listed in Table 1, ZT1 and JB5 were calculated at six construction stages for each condition. The corresponding eigenvalues of the first-order buckling mode were obtained and are presented in Table 6 and Table 7.

Table 6 and Table 7 reveal that the first-order buckling mode eigenvalues of ZT1 and JB5 increase with the increment of the working condition number. For a given condition, the critical buckling load of the light steel frame materials decreases as the construction stage progresses. Specifically, the critical load factor for Stage I is approximately 2.5 times that of Stage VI, indicating that the light steel frame materials are more susceptible to eigenvalue buckling as the number of layers increases. As a result, working condition 1 is deemed the most unfavorable for the structure, and subsequent defect analyses were performed using loads of working condition 1.

### 2.4. Structural Model under the Action of Random Defects

The Monte Carlo method combines the defect-free structure with the most unfavorable working conditions to simulate random defects in the prefabricated light steel frame materials. The defect distribution pattern and amplitude are controlled to add overall and component unit random defects to the finite element model. The eigenvalue buckling analysis is then combined with ZT1, and the first ten orders of buckling modes are obtained when the overall random defects are imposed on the ideal materials. The random defects of the materials are simulated based on Equation (1) using the Monte Carlo method. Here, *m* represents the number of loading conditions, *n* represents the number of selected modes, *c_ij_* is the modal participation coefficient, assumed to be normally distributed, and *φ_ij_* is the *j*th-order normalized buckling mode of the structure for the *i*th condition.

(1) $$\Delta X^{\prime }=\sum_{i=1}^{m}\sum_{j=1}^{n}c_{ij}\varphi_{ij}$$

During the actual calculation process, an amplitude adjustment step aims to make the defect form more similar to the actual materials. The amplitude modulation coefficient, *α*, is defined as the maximum ratio between the value of the structural node’s defective form and the defect’s permissible value. Equation (1) can be transformed into Equation (2) by incorporating the amplitude modulation coefficient. The overall defects of the structure expressed in Equation (2) are brought into the perfected light steel frame materials to impose the initial defects.

(2) $$\Delta X=\alpha \Delta X^{\prime }$$

The initial defects in the component units are mainly initial buckling and residual stresses in the beams and columns. The simulation of random defects in component units is primarily achieved through the initial bending of beams and columns. Furthermore, the distribution forms of defects in different components are independent and randomized. For the initial defects of a single component, the distribution form can be modeled by a sinusoidal half-wave. The initial bending of beams and columns can be uniformly represented by Equation (3), where *δ_max_* represents the defect amplitude, *l* represents the length of the component, and *x* represents the positional coordinate of the point on the component.

(3) $$\delta =\delta_{\max}\sin(\frac{\pi x}{l})$$

Due to the infinite number of possible modes of random encounters between defects in each component, the Monte Carlo method is used to simulate initial defects in components. Assuming that the initial defects of the components follow a normal distribution probability model, the initial bending amplitude of columns and beams is set at 1.05 times the Standard for Design of Steel Structure [42], meeting the requirements with a 95% probability. The Monte Carlo simulation is conducted using Matlab to generate random numbers for the defect amplitude of the distribution model. The sampling values are then randomly arranged to complete the random sampling process. As shown in Equation (4), where *σ*_1_ = *l*/980.

(4) $$P(\delta )=\frac{1}{\sqrt{2\pi \sigma_{1}}}e^{\frac{-{\left(\delta \right)}^{2}}{2\sigma_{1}}}$$

**Figure 5:**
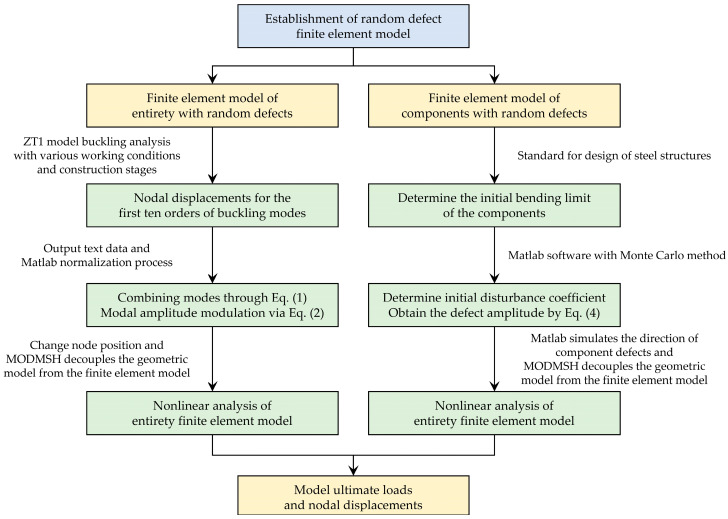
Flowchart for establishing the random defect finite element model.

The process of establishing a random defect finite element model is shown in Figure 5.

## 3. Analysis of Buckling Characteristics under the Effect of Overall Random Defects

### 3.1. Ultimate Load Factor of the Light Steel Frame Materials

**Figure 6:**
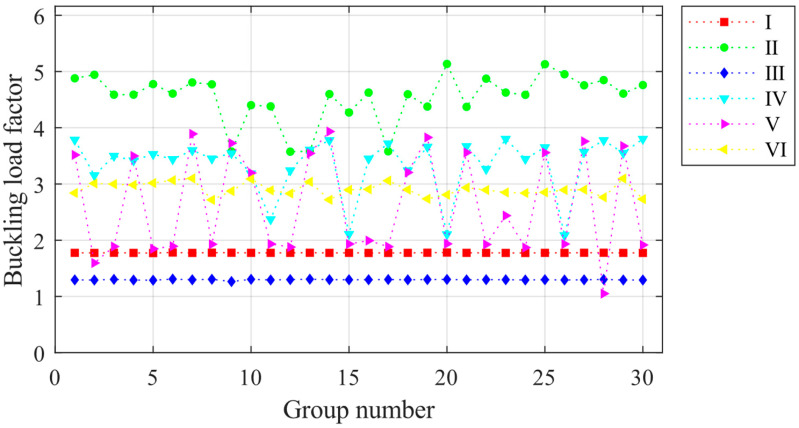
Ultimate load factors for 30 sets of overall random defect models for each construction stage.

The simulation of the overall random defects of the light steel frame materials is based on the eigenvalue buckling analysis of the structure. Nonlinear buckling critical loads are solved using the arc length method. Due to the large number of models, the finite element model is solved in batches using the Ansys Batch program and the RESUME command. Based on the central limit theorem, thirty is determined to be the basic sample capacity of random defects. Thirty simulations with overall defects are performed on the light steel frame materials of ZT1 in each of the six construction stages, and the ultimate load factor is calculated. The results are presented in Figure 6. The structural ultimate load factor is the smallest in construction stage III and the largest in stage II. The ultimate load is not sensitive to the overall defect changes and is relatively small in construction stages I and III, with average values of 1.773 and 1.292, respectively. It can be found in Figure 4 that the structure of the two construction stages has a certain layer of the unfinished stage. The longitudinal stiffness of the light steel frame materials is not uniformly distributed during stages I and III, and the weaker floor of the stiffness controls the limit state. When the structure is in stages II, IV, V, and VI, the data on the structural ultimate load are relatively discrete, and the overall random defect changes greatly affect the load. In these stages, all layers of construction are complete, and the structure does not have a weak position. The ultimate load is changed with the obvious changes in the flexural morphology of the structure due to the overall defect changes.

### 3.2. Light Steel Frame Structural Deformation Properties

The structural deformation performance under varied global random defects during construction is examined by analyzing the ultimate displacements at eight control points of ZT1, subjected to thirty sets of defects across six construction stages. The load sub-steps where the ultimate displacements are located are the load sub-steps where the tangent stiffness matrix singularity is located, i.e., the critical state of the structure for the occurrence of instability. Nodes 1–4 represent the maximum displacement nodes in the first, second, and third floors—and the roof, respectively. Conversely, nodes 5–8 denote the maximum displacement nodes in the columns of the first, second, and third floors—and the roof, in that order. Furthermore, these maximum displacement nodes refer to the positions experiencing the greatest displacement within a column or on a floor at a given construction stage and defect set. Consequently, each model’s specific node locations may vary but consistently symbolize the most hazardous displacements at their respective floors.

**Figure 7:**
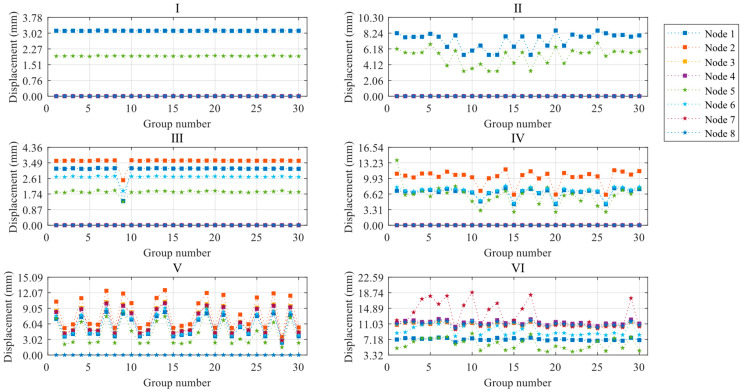
Limit displacement diagrams for control points of ZT1 at each construction stage.

Based on the settings described above, the paper plots the ultimate displacements of control points for each construction stage, as shown in Figure 7, and the average values of limit displacements for control points are shown in Table 8. The ultimate displacements of the nodes increase as the construction stage progresses, with Stages I and III having significantly smaller ultimate displacements than the other stages. This indicates that as the structural completeness increases, the deformation performance of the light steel frame materials improves when it reaches the limit state. Even when the construction of a certain floor is completed, its ultimate displacement is significantly higher compared to the partially completed floor. For instance, Stage II has a higher ultimate displacement than Stage I, while Stage IV has a higher ultimate displacement than Stage III. The degree of variation of node ultimate displacements with defects in construction stages I and III is smaller than in other construction stages. This suggests that stages I and III are less affected by the overall random defects, while the other construction stages are more affected by the variation of defects. Generally, the floor ultimate displacements are larger than the column ultimate displacements for the control nodes of columns and floors on the same floor. This indicates that the overall defects did not affect the trend of the structure to increase the displacements along the height direction. However, construction stage VI is a special case (node 7 compared to node 3). This is because node 7 is located in the layer without floor panels, and compared with construction stage V, there is the role of roof loads. At this stage, node 7 is significantly impacted by overall defects, leading to a significant increase in ultimate displacement compared to node 3.

### 3.3. Two-Factor Analysis of ZT1 Model Calculation Results

The previous calculations indicate that the ultimate load factor may be influenced by two factors: the construction stage and the overall random defects. A two-factor ANOVA without interaction was conducted on the calculated results to investigate the effect of these factors on the ultimate load factor. This analysis assumes that a controlling factor has no significant effect on the results and examines the degree of significance of the effect of each factor on the ultimate load factor by examining the magnitude of the contribution of the variance of the different factors to the total variance.

#### 3.3.1. Two-Factor Analysis of Ultimate Loads

Table 9 shows the two-factor data source analysis results for the ultimate load factor. In the table, SS represents the sum of squares of the data source, df represents the degrees of freedom of the data source, MS represents the mean square of the data source, F represents the ratio of MS, and *p* represents the judgment factor. The resulting *p*-values are 0.273 and 5.05 × 10^−62^. The first *p*-value is for the column factor (defective group number), and since it is greater than 0.05, the original hypothesis is accepted, i.e., there is no significant effect of defective group number on the ultimate load factor. The second *p*-value is for the row factor (construction stage), and since it is much less than 0.05, the original hypothesis is rejected, i.e., there is a very significant effect of the construction stage on the ultimate load factor. This indicates that the construction stage has a much greater effect on the ultimate load than the overall random defects. Still, the overall random defects may have a greater effect on a particular construction stage alone.

**Figure 8:**
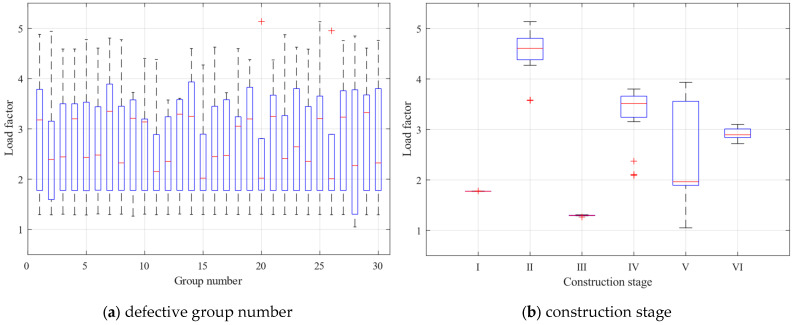
Box plots of the limit load factors for the two influences.

To better observe the bias and tail weight of the calculated limit loads and compare the shapes of the data sets, box plots of the limit load factors for the two influences are presented in Figure 8. The box’s upper and lower blue lines represent the 25% and 75% quantiles of the sample, respectively. The red line in the center indicates the sample’s median, and the end of the dashed line indicates the outer limit of the sample. The shapes of the lines in the box plots in the later section have the same meaning. In Figure 8a, although the distribution of each group of data box plots fluctuates, the overall distribution of samples remains relatively unchanged. This is consistent with the results of the two-factor analysis, indicating that the impact of the overall random defects on the ultimate structural capacity is limited, and its impact is more reflected in the fluctuation of the load factor of a particular construction stage. In Figure 8b, the distribution of each group of data box plots has a significantly different distribution, indicating that the different construction stages of the ultimate load factor have a significant impact. Moreover, the limit load factors are closely distributed in construction stages I and III and more dispersed in other construction stages.

#### 3.3.2. Two-Factor Analysis of Ultimate Displacement of Control Nodes

**Figure 9:**
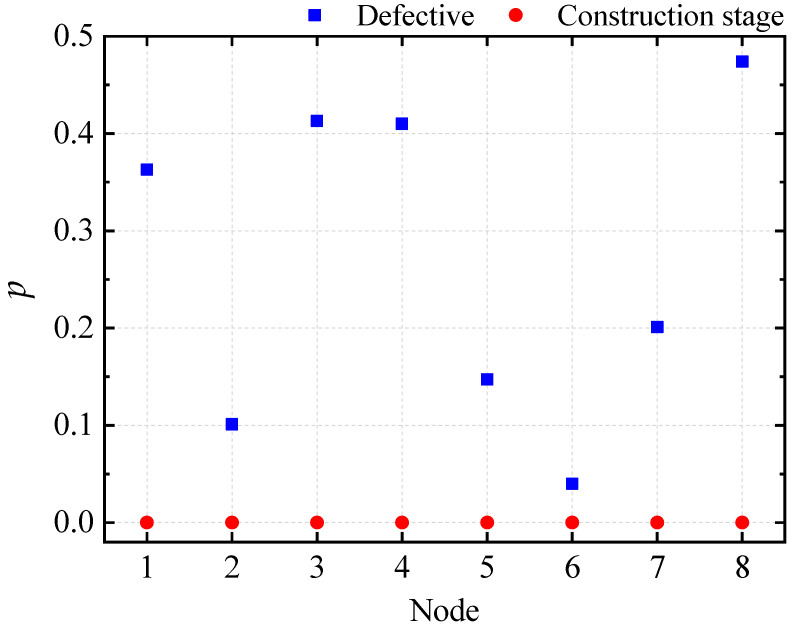
Two-factor analysis plot of the ultimate displacement of the control node for ZT1.

A two-factor ANOVA without interaction was conducted for the overall random defects and the construction stage, and the results are presented in Figure 9. The *p*-value calculated for the construction stage is much less than 0.05 for any node, indicating that the construction phase significantly affects the ultimate displacement of the node, similar to the ultimate load factor. The *p*-value calculated for overall random defects is only 0.04 at node 6 and much greater than 0.05 for other nodes, indicating that the ultimate displacement at node 6 is more affected by the overall random defects than other nodes.

**Figure 10:**
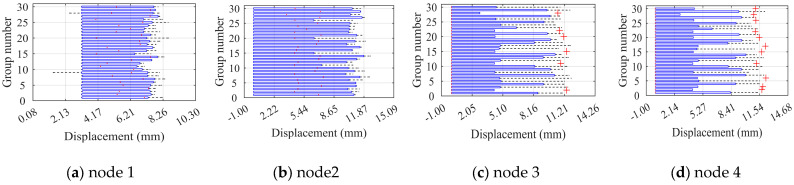

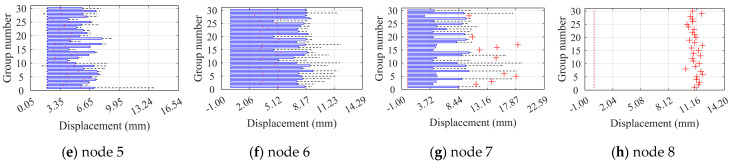
Box plots of ultimate displacements at the control nodes of ZT1 under the overall defects.

**Figure 11:**
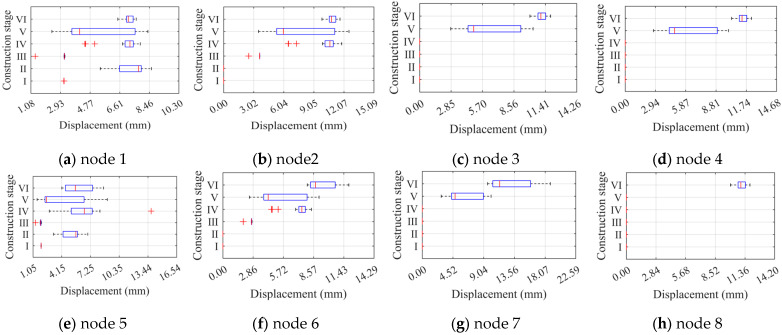
Box plots of ultimate displacements at the control nodes of ZT1 under each construction stage.

To better observe the distribution of node limit displacements, the paper plotted box plots of limit displacements of eight nodes under two different factors separately in Figure 10 and Figure 11. According to the two-factor analysis calculations, node 6 is more affected by the overall defects. Although the distribution of the box plots is not the most obvious change at node 6 in Figure 10, the change of the median point of the sample distributions at node 6 is very noticeable. In contrast, nodes 3, 4, and 7 have a median point of the sample distribution of zero. Combined with the results of the two-factor analysis, it is reasonable to conclude that node 6 is more affected by the overall random defects. Additionally, since node 8 has non-zero displacement only in construction phase VI, the displacement distribution consists of discrete points without box plots. Figure 11 shows that the box plots of node ultimate displacements in the construction stage are significantly dispersed with respect to the overall random defects displacement box plots. This indicates that the construction stage changes significantly affect the node’s ultimate displacements, which is the same as the significant effect of the construction stage on the ultimate load factors. Although the overall random defects do not have a significant effect on the overall node ultimate displacements, the specific node changes in a specific construction stage are still evident.

## 4. Analysis of Buckling Characteristics under the Effect of Component Random Defects

### 4.1. Ultimate Load Factor of the Light Steel Frame Materials

**Figure 12:**
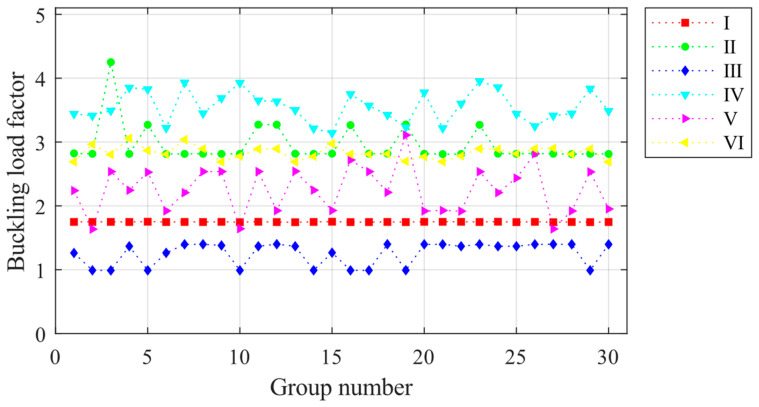
Ultimate load factors for 30 sets of component unit random defect models for each construction stage.

The component unit random defect simulation method was used to simulate 30 sets of component unit random defects for JB5 during construction stages I–VI. The ultimate load factors for each set of defects were calculated, and the results are presented in Figure 12. The ultimate load factor fluctuation is minimal during construction stages I and VI, indicating that changes in component unit defects have minimal influence on the ultimate load factor during these stages. The minimum ultimate load factor of the structure occurred during stage III at 0.9891, while the maximum ultimate load factor occurred during stage IV at 3.951. The maximum ultimate load factor varies significantly across construction stages. However, there is no significant difference between the ultimate load factor under the effect of overall random defects and component unit random defects.

### 4.2. Light Steel Frame Structural Deformation Properties

**Figure 13:**
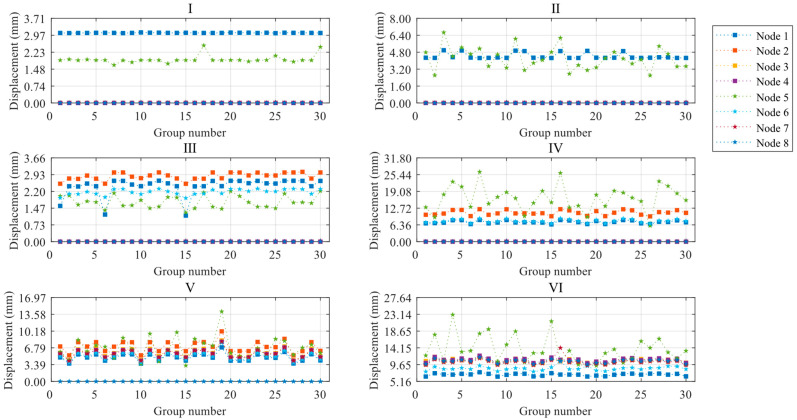
Limit displacement diagrams for control points of JB5 at each construction stage.

The ultimate displacements of eight control nodes of the JB5 were extracted under the effect of 30 sets of component unit defects during six construction stages to analyze the light steel frame materials’ deformation performance. The significance of the load sub-steps and each control node for extracted displacements is the same as previously. The ultimate displacement diagrams for each node are presented in Figure 13. The ultimate displacements of the nodes in construction stages II, IV, and VI are significantly improved compared to their respective previous stages, indicating that the deformation performance of the structure improves with the perfection of the structural system. With the exception of construction stages I and III, the column displacement of the same level is larger than the layer displacement, demonstrating that component unit defects significantly affect the ultimate displacement of the column members. The phenomenon of overall defects only occurs during construction stage VI.

### 4.3. Two-Factor Analysis of JB5 Model Calculation Results

To investigate the effects of component unit random defects and construction stages on the ultimate loads of the light steel frame materials and the ultimate displacements of each control node, and to determine the degree of these effects, a two-factor analysis was performed on the results of JB5 calculations.

#### 4.3.1. Two-Factor Analysis of Ultimate Loads

The two-factor method without interaction was used to analyze the effect of construction stages and component unit random defects on the ultimate load. Table 10 presents the calculation results for the two-factor analysis of the ultimate load. The column factor (defect group number) and row factor (construction stage) have judgment factors of 0.260 and 1.17 × 10^−82^, respectively. The first *p*-value is greater than 0.05, indicating that different component unit random defects have no significant effect on the ultimate load factor. The second *p*-value is much less than 0.05, indicating that construction stages significantly affect the ultimate load factor. The effect of construction stages on the ultimate load is much greater than that of component unit random defects. However, component unit random defects may have a greater effect on a single construction stage. This conclusion is consistent with the findings for overall defects, where the effect of construction stages on the ultimate load factor as a whole is more significant than the effect of defects, but random defects may have more influence on the distribution of ultimate loads for a single construction stage.

**Figure 14:**
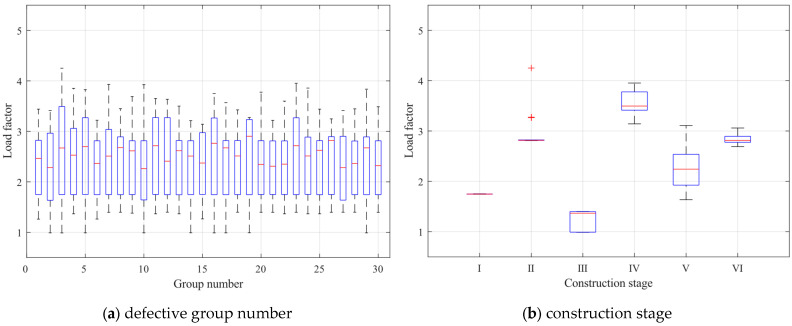
Box plots of the limit load factors for the two influences.

Figure 14 shows the box plots of the ultimate load factor for component unit random defects and construction stages. In Figure 14a, the ultimate load factor fluctuates somewhat with component unit random defects, but the distribution of ultimate loads throughout the construction stages does not show a significant difference. Therefore, the effect of component unit random defects on the distribution of ultimate load factors throughout the construction stages is not significant. The box plot is tighter than that in Figure 8a, and the difference is relatively obvious because the *p*-value obtained from the two-factor analysis of component unit random defects is smaller. In Figure 14b, the construction stages I and II samples are tightly distributed. The construction stage II discrete points are identified as sample anomalies, and the difference in sample distribution between construction stages is significant. Therefore, the ultimate load factor of the light steel frame materials is significantly affected by the construction stage. Compared to Figure 8b, the tightly distributed stages of the ultimate load factor of the structure have shifted from construction stages I and III to I and II, indicating that the structural factor affecting the ultimate load factor has changed from the stiffness difference between the floors to the number of structural floors.

#### 4.3.2. Two-Factor Analysis of Ultimate Displacement of Control Nodes

**Figure 15:**
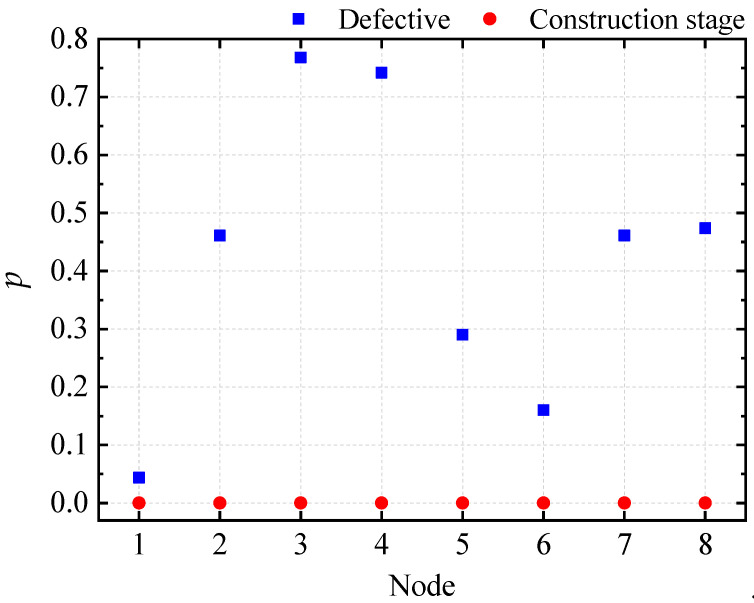
Two-factor analysis plot of the ultimate displacement of the control node for ZT1.

A two-way ANOVA without interaction was conducted on the two factors of component unit random defects and construction stages, and the results are presented in Figure 15. The *p*-value calculated for construction stages at any node was much less than 0.05, indicating a significant effect on the ultimate displacements of the nodes. At node 1, the *p*-value calculated for component unit random defects was also less than 0.05, indicating a significant effect on ultimate displacement only at that node. Combined with Figure 13, we can conclude that the buckling location of the light steel frame materials occurs at the column with the largest displacement on the first floor, indicating that unit random defects significantly affect the critical buckling state of the structure.

**Figure 16:**
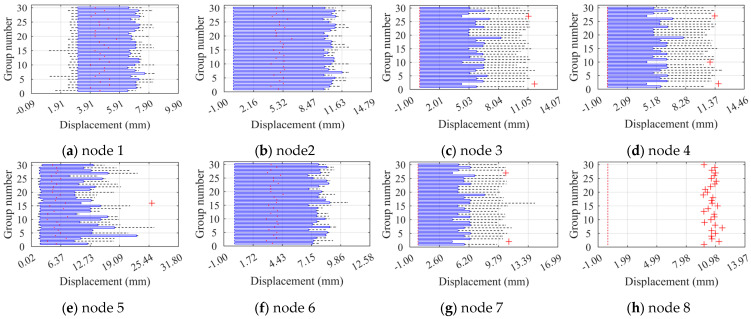
Box plots of ultimate displacements at the control nodes of JB5 under the unit defects.

**Figure 17:**
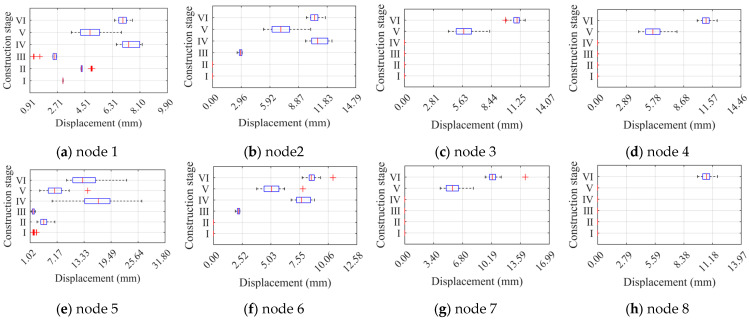
Box plots of ultimate displacements at the control nodes of JB5 under each construction stage.

To better observe the distribution of node limit displacements, we plotted the box plots of the limit displacements of eight nodes separately for two different factors, as shown in Figure 16 and Figure 17. In Figure 16, the change in the locus of the sample distribution of node 1 is more apparent than for the other nodes, indicating that node 1 is more affected by component unit random defects. Compared to Figure 10, the lower limit of the box plot for node 1 with defects changes significantly, and component unit defects widen the distribution of node limit displacements. Similar conclusions are found for node 6. Moreover, since node 8 has a non-zero displacement only at construction stage VI, its displacement distribution consists of discrete points with no box plot. Figure 17 shows that the construction stage change significantly affects the node ultimate displacements, and the length of the box plots is smaller under the pre-construction stage, which is consistent with the significant effect of the construction stages on the ultimate load factor. Compared to Figure 11, the box plots of each node displacement are tighter under component unit random defects, indicating that these defects have less effect on node ultimate displacement.

## 5. Conclusions

This research presents a defect simulation approach suitable for light steel materials during construction. This method is based on the prevailing design specifications for initial defects in light steel framing materials and an analysis of random defects. The Ansys finite element software is employed to evaluate the ultimate load factor and structural deformation of the structure caused by overall random defects and component unit random defects during construction. The findings of the investigation are presented as follows:

(1) Compared to random defects, the construction stage is identified as the primary factor affecting the ultimate load factor (nodal ultimate displacement). When considering random defects as the influencing factor, the distribution of ultimate load factors (nodal ultimate displacements) remains relatively unchanged throughout the construction stage. However, the ultimate load factors of individual construction stages may fluctuate significantly. On the other hand, when the construction stage is considered the influencing factor, the distribution of ultimate load factors (nodal ultimate displacements) varies significantly.
(2) The response of the light steel frame structural ultimate load factor to construction stages and random defects varies depending on the stiffness difference between the layers and the number of layers. When the stiffness difference between the layers is smaller, the ultimate load factor is more affected by overall random defects. Conversely, when the number of layers is lower, the ultimate load factor is more affected by component unit random defects.
(3) When the light steel frame structural integrity is better, the ultimate displacements of both random defects increase significantly, and the deformation performance of the light steel frame materials improves correspondingly. The fluctuation of nodal ultimate displacements is more significantly affected by component unit random defects than overall random defects.
(4) The correlation between the ultimate load factor and the ultimate displacement of the light steel frame varies for different types of defects. There is no correlation between ultimate load factors and ultimate displacements for component unit random defects, whereas a correlation exists for overall random defects. Therefore, it is necessary to differentiate between component unit random defects and overall random defects.
(5) The displacements of the light steel frame tend to develop more rapidly under the action of component unit random defects than under overall random defects. However, the buckling critical loads of the light steel frame are not significantly different between the two types of defects.

Our research aims to offer references and assistance for the construction process of light steel structures. We hope to promote the standardized production of light steel materials, thereby minimizing defects. Additionally, we advocate for implementing temporary bracing during construction, strict adherence to welding procedures, and including specialized analyses to ensure structural stability. In the future, we suggest further simplifying the model, expanding the calculation scale, considering the structural dynamic response, and increasing the structural layers. These measures would enable finite element analysis results to better approximate the most unfavorable limit state of the structure in actual conditions.

## Figures and Tables

**Table 1:** Structural load combination.

Working Condition	Permanent Load	Variable Load	Wind Load	Snow Load
1	1.35	0.7	0.6	0.7
2	1.2	1.4	0.6	0.7
3	1.2	0.7	1.4	0.7
4	1.2	0.7	0.6	1.4

**Table 2:** Standard value for structural load.

Permanent Load		Variable Load	
Name	Standard Value	Name	Usage (Construction) Value
Reinforced concrete	2.5 kN/m^3^	Floor live load	2.0 (0.6) kN/m^2^
Steel	78.5 kN/m^3^	Roof live load	0.5 (0.5) kN/m^2^
C-type light steel keel partition wall	0.54 kN/m^2^	Wind load	0.3 (0.3) kN/m^2^
300 mm cement hollow block	9.6 kN/m^3^	Snow load	0.25 (0.25) kN/m^2^
Hardwood flooring	0.4 kN/m^3^	/	/
Clay flat tile roofing	0.55 kN/m^3^	/	/

**Table 3:** Shapes and dimensions of column and beam (unit: mm).

Column Category	Section Size	Beam Category	Section Size
I-column Z1	259 × 107 × 6 × 9	I-beam B1	250 × 125 × 6 × 9
Square column Z2	75 × 75 × 4.5	I-beam B2	250 × 125 × 3.2 × 4.5
Square roof column Z3	60 × 60 × 2.5	C-beam B3	150 × 50 × 50 × 4.0
		Double C-type roof beam B4	100 × 500 × 20 × 2.5

**Table 4:** Meshing method of the ideal light steel frame materials.

Beam ^1^	Column ^1^	X-Direction Plate ^2^	Y-Direction Plate ^2^
2	1	0.5	0.5
4	5	1	1
6	10	/	/

^1^ Indicates the number of meshing for the corresponding component; ^2^ Indicates the ratio between plate and beam nodes.

**Table 5:** Calculated results after meshing.

Model	Beam ^1^	Column ^1^	X-Direction Plate ^2^	Y-Direction Plate ^2^	Max Displacement	First-Order Buckling Mode
ZT1	4	1	1	1	3.8904	2.8378
JB5	4	5	1	1	3.8966	2.8115

^1^ Indicates the number of meshing for the corresponding component; ^2^ Indicates the ratio between plate and beam nodes.

**Table 6:** Eigenvalues of first-order buckling mode of ZT1.

Working Condition	I	II	III	IV	V	VI
1	7.039	5.502	3.929	3.210	3.196	2.901
2	7.499	5.872	4.223	3.458	3.444	3.128
3	7.856	6.143	4.383	3.584	3.554	3.203
4	7.873	6.155	4.398	3.593	3.577	3.238

**Table 7:** Eigenvalues of first-order buckling mode of JB5.

Working Condition	I	II	III	IV	V	VI
1	6.967	5.406	3.911	3.181	3.168	2.876
2	7.423	5.768	4.204	3.427	3.413	3.099
3	7.776	6.037	4.363	3.552	3.524	3.175
4	7.793	6.048	4.377	3.562	3.546	3.209

**Table 8:** Average value of limit displacements for control points at each construction stage.

Node	I	II	III	IV	V	VI
1	3.136	7.324	3.107	6.945	5.491	7.197
2	0	0	3.575	10.226	8.084	10.875
3	0	0	0	0	6.640	11.026
4	0	0	0	0	6.466	11.354
5	1.925	5.188	1.859	6.355	4.213	5.965
6	0	0	2.690	7.218	5.808	9.433
7	0	0	0	0	6.514	12.982
8	0	0	0	0	0	10.987

**Table 9:** Calculation table for two-factor analysis of ultimate loads.

Source	SS	df	MS	F	*P*
Defective group number	7.055	29	0.243	1.166	0.273
Construction stage	199.911	5	39.982	191.579	5.05 × 10^−62^
Error	30.261	145	0.209	/	/
Total	237.227	179	/	/	/

**Table 10:** Calculation table for two-factor analysis of ultimate loads.

Source	SS	df	MS	F	*p*
Defective group number	1.851	29	0.064	1.179	0.260
Construction stage	107.319	5	21.464	396.395	1.17 × 10^−82^
Error	7.851	145	0.054	/	/
Total	117.022	179	/	/	/

## Data Availability

The data presented in this study are available on request from the corresponding author.
